# RAY-POS: a LIDAR-based assistance system for intraoperative repositioning of mobile C-arms without external aids

**DOI:** 10.1007/s11548-022-02571-w

**Published:** 2022-02-23

**Authors:** Lukas Bernhard, Christopher Völk, Dominik Völk, Florian Rothmeyer, Zhencan Xu, Daniel Ostler, Peter Biberthaler, Dirk Wilhelm

**Affiliations:** 1 Klinikum Rechts Der Isar der Technischen Universität München, Research Group MITI, Munich, Germany; 2 Department of Trauma Surgery, Klinikum Rechts Der Isar der Technischen Universität München, Munich, Germany; 3 Technische Universität München, Chair of Materials Handling, Material Flow, Logistics, Munich, Germany; 4 Department of Surgery, Klinikum Rechts Der Isar der Technischen Universität München, Munich, Germany

**Keywords:** Biomedical imaging, C-arm repositioning, Light detection and ranging (LIDAR), Simultaneous localization and mapping (SLAM), Surgical workflow assistance

## Abstract

**Purpose:**

In current clinical practice, intraoperative repositioning of mobile C-arms is challenging due to a lack of visual cues and efficient guiding tools. This can be detrimental to the surgical workflow and lead to additional radiation burdens for both patient and personnel. To overcome this problem, we present our novel approach *Lidar-based X-ray Positioning for Mobile C-arms* (RAY-POS) for assisting circulating nurses during intraoperative C-arm repositioning without requiring external aids.

**Methods:**

RAY-POS consists of a localization module and a graphical user interface for guiding the user back to a previously recorded C-Arm position. We conducted a systematic comparison of simultaneous localization and mapping (SLAM) algorithms using different attachment positions of light detection and ranging (LIDAR) sensors to benchmark localization performance within the operating room (OR). For two promising combinations, we conducted further end-to-end repositioning tests within a realistic OR setup.

**Results:**

SLAM algorithm *gmapping* with a LIDAR sensor mounted 40 cm above the C-arm’s horizontal unit performed best regarding localization accuracy and long-term stability. The distribution of the repositioning error yielded an effective standard deviation of 7.61 mm.

**Conclusion:**

We conclude that a proof-of-concept for LIDAR-based C-arm repositioning without external aids has been achieved. In future work, we mainly aim at extending the capabilities of our system and evaluating the usability together with clinicians.

## Introduction

### Purpose

Mobile C-arms are an established and widely used means for intraoperative imaging. Due to their maneuverability and degrees of freedom, mobile C-arms play a particularly important role in practical applications, especially for vascular, orthopedic and trauma surgeries. Intraoperatively, the C-arm is usually operated by a non-sterile member of the OR team, such as the circulating nurse. A frequent task for the C-arm operator is to restore previous anatomical views at which X-ray images had been taken earlier, such that the surgeon can monitor the progress or control the quality of treatment. However, the recovery of the corresponding C-arm pose can be rather challenging for several reasons. Firstly, the C-arm may have been moved to a parking position in the meantime—which is commonly done to free space for the surgical team. Consequently, a mere adjustment of the C-arm’s internal degrees of freedom is not sufficient, but the entire 2D spatial pose of the C-arm base within the operating room must be correctly restored. Secondly, the visual appearance of the operating site may have changed and former visual cues, which would have supported realignment of the C-arm, may not be present anymore. For example, this may be due to the installment of tools or coverings, or due to blood leakage caused by surgical manipulations. And thirdly, the C-arm operator must stay clear of the sterile zone and judge the correct positioning of the C-arm’s X-ray axis from a certain distance with limited view onto the surgical site.

As a result, it is common procedure that several X-ray images, so-called *scout images*, are taken—not to extract useful information for the surgical procedure, but merely to retrieve the previous anatomical view. For this, the heavy C-arm needs to be maneuvered repeatedly, which is physically demanding and ergonomically critical, especially for translational corrections. Clearly, this method increases radiation exposure of the patient and the surgical team alike, without providing any therapeutic benefit. That intraoperative C-arm-induced radiation exposure is a relevant problem has been shown by several studies, motivating the need for solutions regarding radiation avoidance [1, 2]. Additionally, the surgical workflow may be delayed considerably by such a trial-and-error approach [3], effectively increasing the stress load of the staff as well as overall costs of the intervention.

To overcome this problem, we herein present our novel approach *Lidar-based X-ray Positioning for Mobile C-arms* (RAY-POS) for assisting circulating nurses during intraoperative C-arm repositioning.

### Related work

Several approaches to the C-arm repositioning problem have been proposed in scientific literature. Some of these rely on the use of infra-red-based tracking or motion capturing systems, where reflective markers are attached to parts of the C-arm and localized by an external stereoscopic infra-red camera [4–6]. While such systems provide a high level of accuracy, there are also considerable drawbacks such as the line-of-sight problem, a rather limited workspace and mandatory external components leading to further cluttering of the already confined OR environment.

Further work has been presented using video-based tracking, where optical markers are attached to the patient anatomy and tracked by a camera attached to the C-arm gantry [7–10]. Thereby, the use of bulky external tracking-cameras mounted on tripods is avoided. While the line-of-sight-problem is still present, it is reduced to the distance between gantry and patient markers. However, depending on the application, preoperative CT scans and additional markers on the patient’s skin are necessary, as well as a cumbersome registration process, demanding additional efforts before or during surgery and thus slowing down the workflow.

Other approaches focus on tracking the C-arm joints, either by attaching sensors [11] or by reading internal joint encoders [12]. While this allows for a precise navigated repositioning of the image plane, the C-arm base is restricted to its initial position. This is a considerable limitation for many practical applications, where the C-arm needs to be moved away from the patient to make room for persons or other equipment.

Haliburton et al. attach a downward-facing video camera to the C-arm base and track its position by means of visual odometry [13]. While this concept avoids many disadvantages of previous approaches and is reported to achieve clinically acceptable accuracy, its applicability to operating room floors with uniform or repetitive textures is in question. The performance of the system is also subject to visual changes of the floor surface, which can be caused by spillage (e.g. blood, colored disinfectants) or objects on the floors (e.g. cables, packaging material of sterile goods).

Unberath et al. propose an approach based on augmented reality (AR), where the C-arm operator is equipped with a head-mounted display (HMD) [14]. The pose of the C-arm can be recorded using infrared sensors and displayed as a virtual object within 3D space, as reference during the repositioning. Even though this approach is quite intuitive for the user, the required AR glasses can be perceived as bothersome and separate the C-arm operator from the environment. Also, switching between different tasks—which is typical for circulating nurses—might be hampered considerably by this. In clinical practice, acceptance of such solutions was quite limited so far.

Further work uses inside-out tracking of the C-arm gantry by means of camera-based simultaneous localization and mapping [15–17]. While accuracy results are not on a par with marker-based tracking, they are promising and certainly acceptable for various clinical applications. However, the suitability of the system for accurately tracking the C-arm position has not been demonstrated yet for long movements of the C-arm base. It is therefore unclear, whether an image pose can be restored after the C-arm has been completely moved away from the OR table. Moreover, the approach requires the surgeon to wear an AR HMD which is an additional burden and raises concerns regarding sterility.

Lastly, different laser aiming devices have been proposed [18, 19] and are commonly provided in modern C-arm models. When working with these aids, the user needs to rely on external landmarks of the patient or the sterile covering. During surgery, these landmarks are subject to frequent change, in which case they cannot be used as reference anymore or may even misguide the user. In practice, the visibility of the laser markers is also frequently compromised by the bright OR lamps illuminating the surgical site. The fact that the C-arm operator must stay away from the sterile zone around the operating table leads to an insufficient view on the surgical site and further complicates the repositioning procedure.

Since above approaches either require substantial overhead for the surgical workflow (external components, setup times etc.) or suffer from other individual drawbacks, there is a need for a simple and reliable solution without any external components. With our RAY-POS concept, we therefore aim at providing a compact, easy-to-use system for C-arm repositioning that does not significantly increase workload for the surgical team.

## Materials and methods

### Concept

Our system provides an intuitive *graphical user interface* (GUI) for recording the C-arm’s current pose within the OR environment and, once requested by the surgeon, assists the C-arm operator in manually guiding the C-arm back to this exact pose. For accomplishing this, our system does not require any external components (e.g. patient markers, cameras or head-mounted displays), which is why the entire system can be easily integrated into the C-arm itself or attached to its casing. We aimed at keeping the overhead for the user at an absolute minimum by reducing the interaction to a single button press, followed by simple on-screen instructions. From an algorithmic standpoint, the localization is based on *simultaneous localization and mapping* (SLAM). Such algorithms use different sensor modalities—in our case 2D *light detection and ranging* (LIDAR) sensors and an *inertial measurement unit* (IMU)—to construct a map of the environment (*mapping*) and, at the same time, calculate the current pose within the map (*localization*). 2D LIDAR sensors measure the distance to surrounding obstacles (e.g. walls) by rotating a pulsed laser beam and detecting the reflected light in an adjustable number of directions within the measurement plane. Using methods such as triangulation or time-of-flight, the distance can be calculated for each direction. Inertial measurement units are used to measure accelerations and angular velocities, which can be used to further improve SLAM quality. Additional information on SLAM can be found in [20].

RAY-POS is meant to complement the *joint angle* storage feature already offered by many modern C-arm products by providing the means for storing the *room pose* of the C-arm base as well. Thereby, a global localization of the image plane within the entire operating room is achieved, even if the C-arm base is moved. We investigated the suitability of three open-source SLAM algorithms for this purpose and compared five different sensor attachment positions as well as external disturbances (movement of persons, movement of the C-arm gantry) regarding their influence on localization accuracy and stability.

### Graphical user interface

**Fig. 1:**
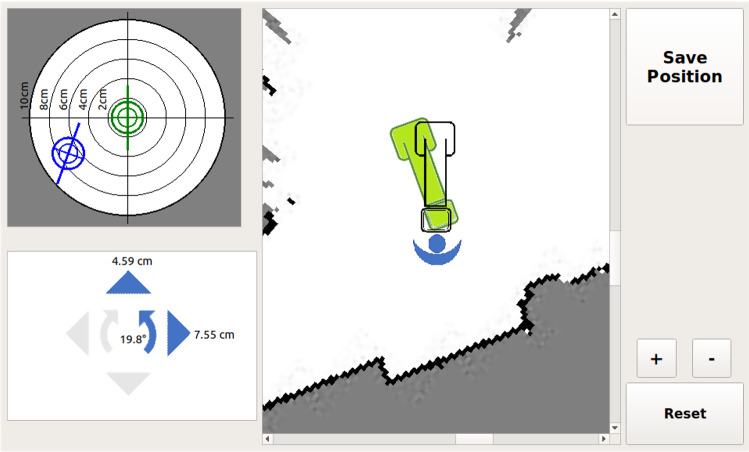
An overview of the RAY-POS graphical user interface is shown. The GUI consists of a map of the surrounding (center), a target (upper left corner), a direction indicator (lower left corner) and buttons for saving the C-arm position, zooming in/out and clearing the saved position (right side)

When designing RAY-POS, we aimed at providing a very simple and intuitive user experience, such that virtually no training is required to use the system. To enable this, a graphical user interface was developed that provides all necessary information for the navigation process. As shown in Fig. 1, the GUI consists of a *map*, a *target*, and a *direction indicator*. The map provides a top-down view of the operating room and is centered on the current pose of the C-arm (according to SLAM data). The C-arm itself is displayed as an outlined symbol. The current pose can be saved using a button and is then displayed as a green symbol within the map. Thus, the symbol can be used as a rough target in case the saved pose needs to be restored. The target and the direction indicator can be used for fine adjustment and become activated as soon as the C-arm is less than 10 cm away from the saved position.

### Hardware

**Fig. 2:**
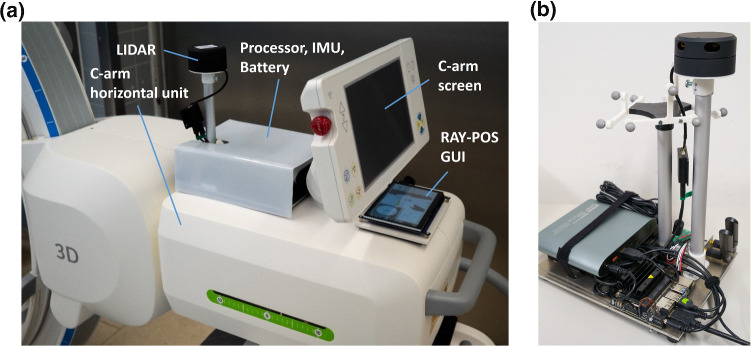
**a** The attachment and components of the RAY-POS system to the C-arm are shown for one of the investigated LIDAR positions (position H20, as explained in the following). The main platform of the system is mounted on top of the horizontal unit of the C-arm and the RAY-POS GUI is displayed by an additional screen below the C-arm’s user interface. **b** The installation of the NDI tracking markers, as used during the localization tests, is shown

Since we aimed at keeping the overall cost of our system low, we selected affordable off-the-shelf components for building our prototype. Data input for SLAM was provided by combinations of 2D LIDAR and IMU sensors, in particular the models *YDLIDAR G2* and *G4* (Shenzhen EAI Technology Co, Shenzhen, China) and *ISM330DLC* (STMicroelectronics, Amsterdam, The Netherlands). A touch LCD display was used for presenting the RAY-POS GUI to the C-arm operator. As main processor and communication hub, the compact single-board computer Jetson Nano (NVIDIA Corporation, California, USA) was used. Using 3D-printed adapters and other aids, all RAY-POS components were reversibly attached to a *Cios Spin* C-arm (Siemens Healthcare GmbH, Erlangen, Germany), as shown in Fig. 2.

As ground truth for evaluating the localization accuracy of RAY-POS, we used the optical instrument tracking system *Polaris Vega* (Northern Digital Inc., Waterloo, Canada). The system allows for a precise localization of the infrared-reflecting trackers within the 3D measurement volume of the camera with a root mean square error of 0.15 mm [21]. The NDI tracking spheres (markers) were arranged in 3 vertical outward-facing facets with 3 markers each, to enable a 360 degree tracking of the c-arm within a horizontal plane parallel to the ground. The installation position of the markers is shown in Fig. 2.

### Data acquisition and SLAM

The entire data processing infrastructure was implemented using the *Robot Operating System* (ROS) running on the Jetson Nano. A dedicated ROS node for the optical tracking system was implemented. Using the ROS tool *rosbag*, we were then able to record the different data streams (LIDAR, IMU, optical tracking) collectively and in a time-synchronized fashion, e.g. to later compare the performance of different SLAM algorithms on one and the same data set. However, for “online” end-to-end operation of the navigation system, SLAM needs to run directly on the Jetson Nano, receiving the sensor data as immediate input.

We conducted a review of 13 open-source SLAM algorithms to identify suitable candidates for our purposes. Based on several criteria (required sensor inputs, establishment, maintenance state and community feedback),^1^ we selected three algorithms, *gmapping*^2^ (GM)*, hector_slam*^3^ (HS) and *Google Cartographer*^4^ (GC)*,* for further investigation.

### Experiments

We conducted two sets of experiments to optimize and evaluate the performance of RAY-POS. First, a preliminary assessment of the absolute localization accuracy of several combinations of sensors, algorithms and other parameters was made to gain knowledge about different influences and to identify promising setups. In the following, we will refer to these experiments as *localization tests*. Based on these findings, two promising combinations of LIDAR position and SLAM algorithm were selected for further investigation. The performance of these was evaluated in an online end-to-end test of the RAY-POS system with a focus on repositioning accuracy, i.e. the ability to precisely lead back to a previously saved position within the operating room. These experiments are referred to as *repositioning tests*. All experiments took place at an experimental, but fully equipped operating room at *Klinikum rechts der Isar* (Munich, Germany). To ensure that the system was exposed to realistic sensor data during the experiments, the environment was set-up to resemble an actual surgical intervention as closely as possible, including an OR table, a patient dummy, surgical draping, OR lights, an anesthesia workstation, the c-arm and its corresponding cart with screens, instrumentation tables and various other equipment, which we did not use, but is commonly present within OR environments (laparoscopic tower, storages etc.).

**Fig. 3:**
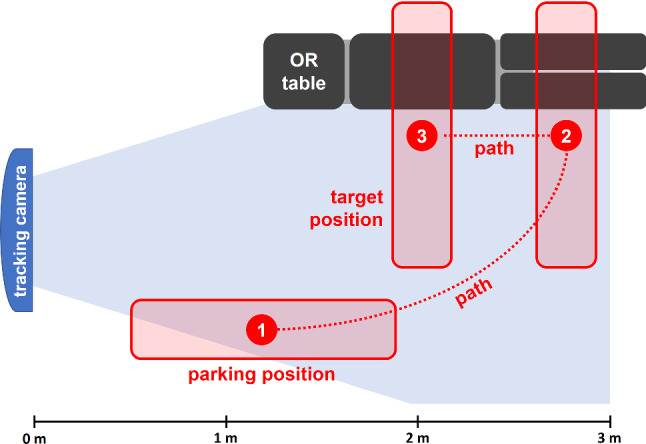
Schematic illustration of the path traversed by the C-arm during the localization tests. The path starts at a parking position next to the OR wall (1) and continues in a curved line until the distal end of the patient phantom on the OR table is reached (2). The C-arm is now moved in parallel to the OR table until the target position over the surgical site is reached (3)

Based on common application scenarios (e.g., posterior instrumentation in spine surgery), a typical intraoperative path for the C-arm was chosen, which is shown in Fig. 3. For the localization tests, the optical tracking system was positioned such that the entire C-arm path was enclosed by the measurement volume of the camera. The C-arm was then moved along the path and back to the parking position, while recording data from both the RAY-POS sensors and the optical tracking system.

**Fig. 4:**
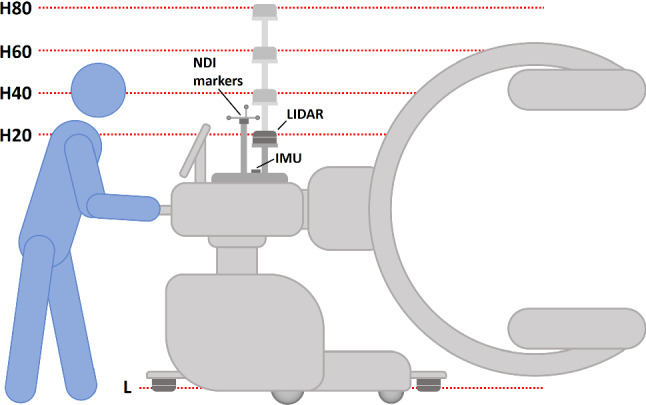
Different alternative positions at which we attached LIDAR sensors to the C-arm are shown. At the lower position (L) two LIDAR sensors were used in conjunction, while only a single sensor was used at each of the upper positions (H20 to H80). The figure also shows the attachment positions of the IMU and the NDI markers (schematically)

To be able to investigate different factors influencing SLAM quality, we created several types of data sets by varying the parameters *LIDAR position, presence of moving objects* and *duration*. Depending on the height at which a LIDAR sensor is attached to the C-arm, a different cross-section of the OR environment is visible within the scan data and different combinations of occlusions (C-arm operator, C-arm gantry, C-arm screen) may be present. The different positions, occlusions and sensor combinations are illustrated by Fig. 4. The lower LIDAR position is referred to as *L*, the upper LIDAR positions are labeled by an *H* followed by the height of the sensor relative the C-arm’s horizontal unit in cm (e.g., H20: LIDAR mounted 20 cm above horizontal unit). In the following, we will refer to combinations of LIDAR position and SLAM algorithm using the @-shorthand, e.g. GM@H20.

We also investigated the influence of moving objects on localization accuracy. During the movement of the c-arm from parking to target position, two persons were walking within the sterile zone at the other side of the OR table to simulate the presence of a surgeon and a scrub nurse. After maneuvering the C-arm to the surgical site, the angular and orbital angles of the C-arm gantry were manipulated, which (depending on the sensor position) affects the LIDAR data. From the standard position of 0°, the angular angle was first changed to + 25°, then to − 25° and then back to 0°. After that, the orbital angle was changed from 0° to 90° and back to 0°. Such manipulations are typically made when adjusting the image plane, e.g. during spine surgery.

To determine whether localization accuracy decreases over longer periods of time for certain SLAM algorithms, we gathered additional long data sets, where the path was traversed for five consecutive times, instead of only once.

In a post-processing step, each data set was played back in real-time to feed the data stream into the three pre-selected SLAM algorithms (i.e., GM, HS and GC). The resulting trajectories where then compared to the optical tracking data, which we assumed to represent the true position of the C-arm over time. We determined the *localization error* over time by calculating the Euclidian distance between both trajectories for each time step. According to the Shapiro–Wilk test, the localization errors were not normally distributed, which is why non-parametric methods were used. We calculated common measures of descriptive statistics such as median, first and third quartile, mean absolute deviation and maximum localization error to rank all combinations of LIDAR positions (L, H20, H40, H60, H80) and SLAM algorithms with respect to these measures. For pairwise comparison of the different combinations, the Mann–Whitney test was used, while compensating for the multiple testing problem using Bonferroni correction, where appropriate.

Based on our long data sets, we investigated how the different combinations perform over longer periods of time, e.g. to identify drift effects. For that, we plotted the error curve over time and fitted a straight line to visualize the overall tendency of the data. Based on that we tried to identify trends (in particular, an increasing error over time). The slope of the linear fit was used for comparing different combinations regarding their tendency.

**Fig. 5:**
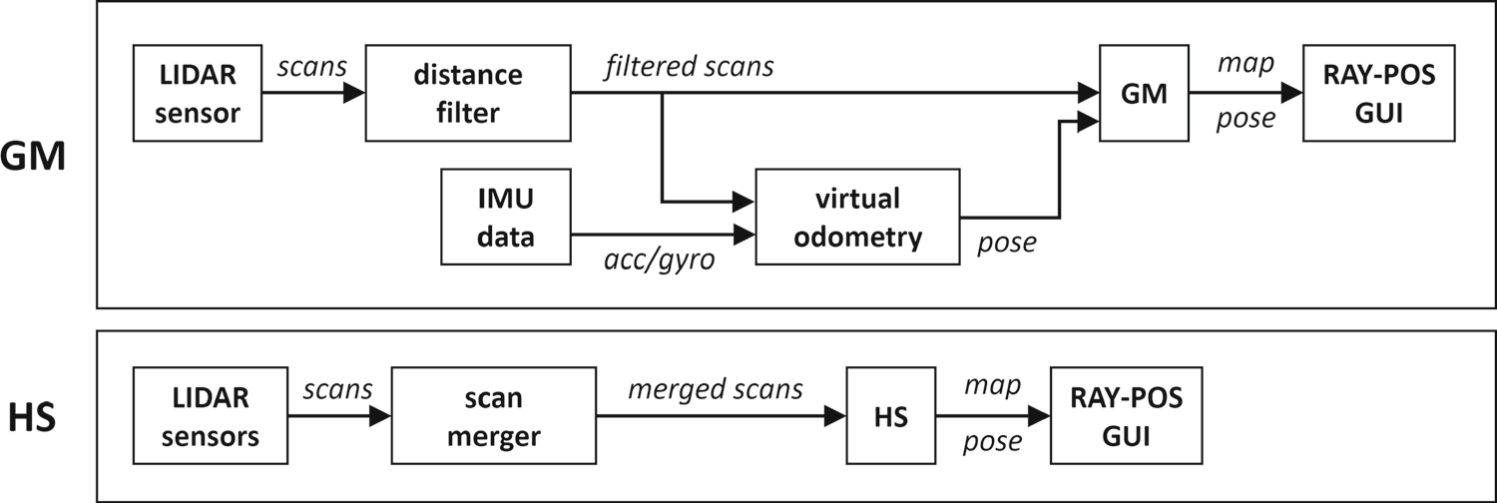
Online end-to-end data processing pipeline for GM and HS. GM receives the input of a single LIDAR sensor. Distances closer than 1 m are filtered out (ROS package *laser_filters*, filter type *LaserScanBoxFilter* (https://wiki.ros.org/laser_filters)) to deal with the dynamic blockings introduced by the C-arm gantry. Since GM requires odometry input, we used a virtual odometry signal (ROS package *laser_scan_matcher* (https://wiki.ros.org/laser_scan_matcher)) based on IMU and LIDAR data as a substitute. For HS, we used a LIDAR scan merger (ROS package *ira_laser_tools*, type *laserscan_multi_merger* (https://wiki.ros.org/ira_laser_tools)) to combine the scans of the front and back LIDARs. A distance filter was not required for HS, since the static blockings of the C-arm base were addressed by limiting the scanning angle of the LIDARs

Based on the outcome of the localization tests, we selected two alternative combinations—one among the upper (H20-80) and one among the lower (L) combinations—for assessing the repositioning accuracy of the entire RAY-POS system in an end-to-end (“online”) fashion. The data processing pipelines for GM and HS are shown in Fig. 5. For both selected combinations, 30 data points were gathered using the C-arm’s laser crosses and coordinate paper. According to the Shapiro–Wilk test, x- and y-coordinates of the resulting *repositioning error*^5^ were normally distributed for both combinations, which is why parametric methods have been used for the statistical analysis. Levene’s test was used for testing variance homogeneity. Based on the standard deviations in x- and y-direction (${\sigma }_{x}$ and ${\sigma }_{y}$), the *effective standard deviation* ${\sigma }_{e}$ was calculated as follows:

$${\sigma }_{e}=\sqrt{{\sigma }_{x}^{2}+{\sigma }_{y}^{2}}$$

## Results

### Localization accuracy

Average results for data sets representing short trajectories *without* disturbances are given in Table 1 (10 data sets for each combination of LIDAR position and SLAM algorithm). According to the measures presented, the GM algorithm at H40 yielded the best results among all upper LIDAR positions (H20-80), with a median of 11.77 mm, a maximum position error of 45.43 mm and a mean absolute deviation of 7.64 mm. To analyze the significance of this observation, we conducted pairwise Mann–Whitney tests with all other upper combinations of SLAM algorithms and LIDAR positions, while using a Bonferroni-corrected significance level of $\alpha =0.0042$. By this, we were able to confirm a significantly better performance of the GM@H40 combination for all pairs (*p* < 0.0003), except when comparing with combination GM@H60 (*p* = 0.076). Regarding the lower LIDAR position (*L*), we found HS to be yielding significantly better results (median: 15.52 mm, maximum position error: 72.44 mm, mean absolute deviation: 9.74 mm) than GM (*p* < 0.0001; $\alpha =0.025$), while GC failed to provide usable results.

**Table 1:** Localization accuracy of pre-selected SLAM algorithms for short trajectories without disturbances is summarized

SLAM algorithm	LIDAR position	Q1	Median	Q3	Max	MAD
GM	H20	10.60	18.62	27.69	78.65	10.16
	H40	6.04	11.77	19.47	45.43	7.64
	H60	6.37	12.46	20.00	46.67	7.75
	H80	6.45	13.03	20.60	52.94	8.15
	L	15.32	31.15	47.51	494.91	43.43
HS	H20	17.46	25.06	34.50	80.93	10.74
	H40	13.21	19.66	27.84	61.28	9.05
	H60	12.33	17.98	25.33	69.89	8.96
	H80	11.17	19.01	26.56	62.86	9.46
	L	9.11	15.52	23.14	72.44	9.74
GC	H20	16.97	25.25	34.88	92.36	10.65
	H40	9.54	15.80	22.99	54.95	8.06
	H60	8.41	14.40	24.39	69.58	10.63
	H80	11.09	18.37	25.79	56.87	8.29

The given parameters describe the distribution of the SLAM position error with respect to the ground truth (optical infra-red tracking) for the investigated algorithms GM, HS and GC. First quartile (Q1), median, third quartile (Q3), maximum position error (Max) and mean absolute deviation (MAD) are given in [mm]. Lower values indicate a better performance. Since GC did not provide usable results for position L, no results can be given for this combination

Results for short trajectories *with* disturbances are given in Table 2 (10 data sets for each combination of LIDAR position and SLAM algorithm). Again, the two combinations GM@H40 and GM@H60 yielded the best results, with H40 performing slightly better regarding maximum position error (49.32 mm) and mean absolute deviation (4.98 mm), and H60 yielding a lower median (6.65 mm). Both combinations are significantly better than the remaining upper combinations of LIDAR positions and SLAM algorithms (*p* < 0.0001; $\alpha =0.0056$).

**Table 2:** Localization accuracy for short trajectories with disturbances

SLAM algorithm	LIDAR position	Q1	Median	Q3	Max	MAD
GM	H20	11.71	16.75	21.47	76.53	6.29
	H40	4.68	7.78	11.65	49.32	4.98
	H60	3.30	6.65	13.86	55.30	6.65
HS	H20	22.84	27.71	32.94	61.66	7.08
	H40	17.34	22.75	28.01	64.80	7.38
	H60	12.35	16.15	19.43	53.75	5.17
GC	H20	14.26	20.38	27.14	80.24	9.39
	H40	5.34	8.74	13.38	62.75	7.51
	H60	15.82	24.86	39.12	74.32	13.72

**Fig. 6:**
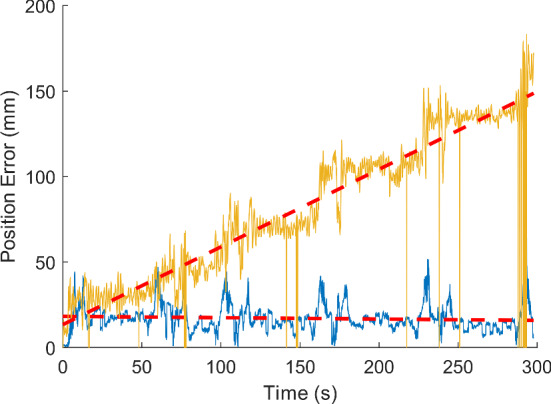
Plot of localization errors yielded by GM (blue graph) and GC (yellow graph) for the same data set (H40 with disturbances, 5 traversals). For both data sets, the linear fit is visualized as a dashed red line

Results for the long data sets are summarized in Table 3 (one data set for each combination of LIDAR position and SLAM algorithm). For each combination, the slope of the linear fitting is given, as a measure for the long-term behavior of the localization error over the course of five traversals. Most notably, GC was subject to a considerable amount of drift over time resulting in slope values one or even two orders of magnitude higher than those of the other algorithms. An example of this effect is shown in Fig. 6, where GC and GM yield very different results for the same data set.

**Table 3:** The slope of the straight line fitted to the localization error over time is given in [mm/s] for each combination of SLAM algorithm and LIDAR position as a measure for the long-term tendency of the error

SLAM algorithm	Disturbances present	H20	H40	H60	H80	*L*
GM	No	0.0112	0.0244	0.0070	− 0.0038	0.1693
HS		− 0.0086	0.1241	0.1206	0.0525	0.0631
GC		0.6482	0.8159	0.8236	0.3984	–
GM	Yes	− 0.0221	− 0.0079	− 0.0121	–	–
HS		0.0221	0.0041	0.0533	–	–
GC		0.3980	0.4548	0.3543	–	–

Values close to zero indicate a good performance. The column *Disturbances Present* indicates whether the data was subject to external disturbances (Note that H80 and *L* were excluded, since H80 was too high to be disturbed by moving persons or the C-arm gantry, and *L* was too low to be disturbed by the C-arm gantry and was shielded from moving persons by the sterile coverings of the OR table). Refer to Fig. 6 for a visualization of the linear fit

**Fig. 7:**
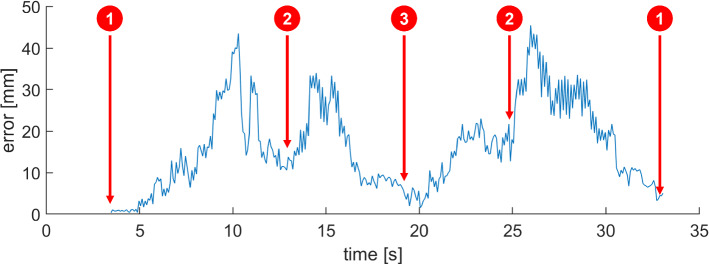
Plot of the localization error over time for one of the GM@H40 data sets. The numbers indicate the C-arm position as defined by Fig. 3

We have also observed that fast movement of the C-arm temporarily increases the localization error as compared to phases of slow or no movement. This is illustrated by Fig. 7, where the localization error over time is plotted for an exemplary traversal of the path. As can be observed, the error is highest for the two phases between C-arm positions 1 and 2 (see Fig. 3), where the C-arm velocity was highest. On the other hand, the error has its smallest values around the start and end position (1) as well as directly at the final position (3), where C-arm movement was briefly paused. We further comment on implications of this effect in the Discussion section.

### Repositioning accuracy

**Fig. 8:**
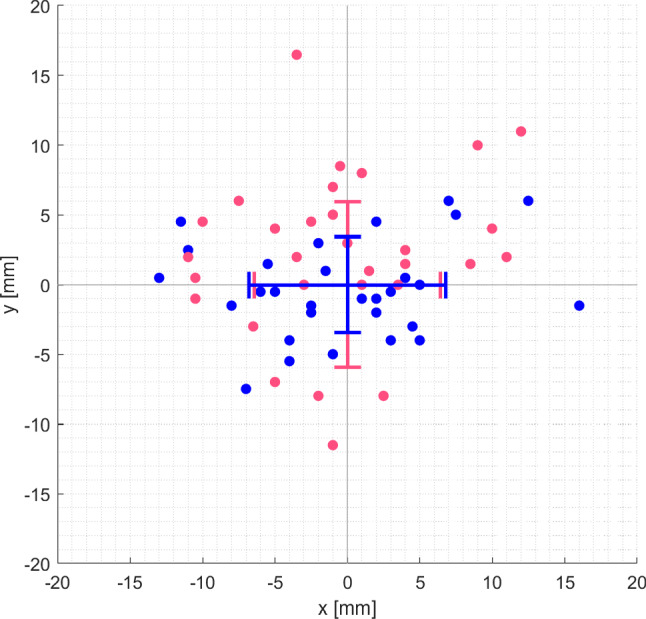
The plot shows the results of the repositioning tests. Deviations from the target position (0, 0) for GM@H40 (blue) and HS@L (red) are displayed. The crosses mark the respective standard deviations

As will be elaborated in the discussion section of this paper, we selected GM@H40 and HS@L for further assessment regarding accuracy of repositioning. For both setups, the resulting deviations from the target position that have been recorded during the repositioning tests are shown in Fig. 8.

According to the Shapiro–Wilk test, the data is normally distributed in x- and y-direction. Descriptive statistics are given in Table 4. With a maximum repositioning error of 16 mm and an effective standard deviation of 7.61 mm, GM@H40 performed slightly better than HS@L, however the difference in variance is not significant according to Levene’s test (x-direction: p = 0.65; y-direction: *p* = 0.06).

**Table 4:** Descriptive statistics for the repositioning tests are summarized

SLAM algorithm	LIDAR position	*n*	Max	*σ*_*x*_	*σ*_*y*_	*σ*_*e*_
GM	H40	30	16	6.80	3.42	7.61
HS	*L*	30	16.5	6.44	5.92	8.75

Sample size (*n*), maximum repositioning error (Max in [mm]), standard deviation along x-axis (*σx* in [mm]), standard deviation along y-axis (*σy* in [mm]) and effective standard deviation (${\sigma }_{e}$ in [mm]) are given

When comparing the variance of x- and y-direction for each algorithm separately, we have observed no statistically significant difference for HS@L (*p* = 0.46). However, GM did show a significantly different performance between x- and y-direction (*p* = 0.0009).

## Discussion

According to the results presented in the previous section, the GM algorithm showed superior performance for upper LIDAR positions. Since no significant difference between H40 and H60 positions was observed, the lower H40 position was selected for the repositioning tests due to being a more compact overall solution. Additionally, we chose to select an alternative setup for the *L* position, since—from a product development standpoint—an integration of LIDAR sensors into the front and back of the C-arm base might be considered more seamless than a pole on the horizontal unit. Also, the horizontal unit—and thus the LIDAR sensor—can usually be moved relatively to the base, which must be compensated for during localization. Since the HS algorithm yielded best results for the lower position, HS@L was chosen. This effect might be explained by the ability of HS to better handle the LIDAR signal at the L position, which is merged from two LIDAR sources. This process introduces an additional error within the signal chain. Other individual differences of the two positions, e.g. regarding the characteristics of the obstacles observed at the different heights, might also be of influence.

According to Table 1, varying the LIDAR height between H40 and H80 did not have a major impact on localization performance across all SLAM algorithms (1.26 mm maximum difference in median for GM, 1.68 mm for HS and 2.57 mm for GC), which should not make a discernable difference in practical application. However, results for H20 indicate a slightly inferior performance (median increased by 5.59 mm for GM, 5.40 mm for HS and 6.88 mm for GC), which might be explained by the additional limitation to the angle of view introduced by the C-arm’s display. Therefore, we recommend an installation of the LIDAR sensor above H20 for optimal SLAM performance, at least for this C-arm model. On the other hand, we argue that it should be possible to achieve acceptable results with H20 as well—or with an even lower placement of the LIDAR sensor directly on the horizontal unit. The latter would provide a more seamless integration of RAY-POS, without any elements protruding considerably from the regular C-arm casing.

As described in the Results section, we observed that the SLAM algorithms are sensitive to fast movement of the C-arm, while the localization error tends to quickly decrease again after this movement stops. As a result of this, data sets containing longer phases of slow movement—and thus more data points with small error values—*seem* to perform better when looking at descriptive statistical parameters (such as the median). For example, this can be observed when comparing data sets with and without disturbances. Additional time was spent for manipulating the angular and orbital angles of the c-arm, while the c-arm base is not moved at all during this procedure. Thus, a direct comparison of the results for data sets with and without disturbances is only sensible with regard to the maximum error—where no major increase has been observed.

Regarding the end-to-end repositioning accuracy, we found that both GM@H40 and HS@L worked equally well. The differences in variance observed between accuracy in x- and y-directions for GM@H40 could be due to a weaker performance of GM regarding z-rotation accuracy. Since the center of rotation is located on the c-arm base, rotational errors introduce translations at the center of the image plane, due to the distance between base and image plane center acting as a lever arm. The fact that the observed repositioning errors were smaller than the median localization error can be explained by C-arm velocities being slow during the fine-positioning close to the surgical site. With reference to the insights gained by our localization tests, this situation is associated with a low average error, compared to phases of fast C-arm movements.

While better accuracies have been achieved by other methods presented in the literature—most notably systems based on optical tracking—our results are promising and provide an important proof-of-concept for LIDAR-based C-arm navigation. In relation to the total dimensions of the X-ray image (e.g., 300 × 300 mm for the Cios Spin), the distribution of the repositioning error is small, and thus should not carry any weight for most practical applications. It must also be considered that inevitable patient and organ movements can contribute to the repositioning error, which limits the achievable accuracy for approaches without patient-tracking from the outset. Our work was motivated by the clinical need for an uncomplicated solution without requiring any external components, additional preparation steps or special requirements regarding the OR environment. All these demands are perfectly fulfilled by our approach, while providing the additional benefit of low manufacturing costs and a straight-forward integration into C-arm designs. We believe that exactly these aspects make all the difference when it comes to translation of research prototypes into real-world application. Nonetheless, we are confident that accuracy can be further improved using higher-quality sensors.

Special consideration must be given to swivel rotations of the C-arm. While the localization of the C-arm base is not influenced by swivel movements in cases where the LIDAR sensors are directly attached to the base (L position), there are limitations of the current RAY-POS prototype for upper LIDAR positions (H20-80). As of now, the localization is not able to discriminate between rotations of the entire C-arm base and internal rotational due to swivel. As a possible solution, one could use measurements of the current rotation of the swivel joint for compensation (e.g. using interfaces to the internal joint encoder or by means of externally attached sensors).

Regarding limitations of our study, we must point out that the investigations regarding disturbances caused by moving objects can only be seen as a first step. There may be considerably more persons present during surgical interventions and there may be other moving objects, such as screens, tables, devices etc. The limited tracking volume of the NDI camera used as ground truth for the localization tests can be seen as a further limitation. Due to this, the length of the C-arm path was confined to the tracking volume and we can therefore not make any statements regarding the localization accuracy of SLAM algorithms for longer paths. Moreover, it is desirable to further evaluate our system together with C-arm operators to gain insights into usability aspects and compare the performance to traditional non-navigated C-arm repositioning. For now, however, we chose to focus on the benchmarking of different LIDAR positions and SLAM algorithms, which has provided many novel insights and should provide a strong foundation for further work regarding LIDAR-based C-arm localization.

## Conclusion

Within this paper, we presented our approach RAY-POS for intraoperative C-arm repositioning without external aids. A systematic comparison of different SLAM algorithms and LIDAR attachment positions has been conducted to maximize localization accuracy. Based on these results we selected two combinations for further assessment regarding repositioning accuracy. Both options yielded promising results and demonstrated the feasibility of LIDAR-based C-arm localization. Furthermore, we presented the graphical user interface of RAY-POS that aims at guiding the C-arm operator in an intuitive manner.

In future work we mainly aim at integrating new features into our system, e.g. to enable the user to save multiple positions and define offsets towards saved positions for relative navigation. Also, the GUI experience could be further enhanced by matching a pre-existing map to the one provided by the SLAM algorithm. Thereby, a better comprehensibility and overall user experience could be achieved.

To conclude, a proof-of-concept for LIDAR-based C-arm repositioning without external aids has been presented. While the results are very promising, further improvements and studies need to be conducted to push the system towards practical application.

## Footnotes

1 In total, 13 algorithms where compared using a weighted score. For each criterion, the algorithms were rated by granting 0–2 points. Regarding “sensor inputs”, algorithms were preferred that were able to use both modalities provided by the RAY-POS system (LIDAR and IMU). “Community feedback” was rated based on positive/negative reviews provided by the community. Regarding “establishment”, the relative amount of discussions, mentions and reports that we have found during our investigations were used an estimate. “Maintenance state” was rated based on the supported ROS version and the maintenance status reported by ros.org. The final score was calculated for each algorithm by adding the points in each category. Based on the results, the three algorithms GM, HS and GC were selected for the purpose of this paper.

2 http://wiki.ros.org/slam_gmapping.

3 http://wiki.ros.org/hector_slam.

4 https://google-cartographer.readthedocs.io/en/latest/.

5 The rotation was recorded as well, however, the deviation from the initial rotation was barely measurable. Therefore, we chose to neglect the rotation error during further analysis.
